# Chorioallantoic Membrane Assay at the Cross-Roads of Adipose-Tissue-Derived Stem Cell Research

**DOI:** 10.3390/cells12040592

**Published:** 2023-02-12

**Authors:** Dmytro Oliinyk, Andreas Eigenberger, Oliver Felthaus, Silke Haerteis, Lukas Prantl

**Affiliations:** 1 Department of Plastic, Hand and Reconstructive Surgery, University Hospital Regensburg, Franz-Josef-Strauss-Allee 11, 93053 Regensburg, Germany; 2 Institute for Molecular and Cellular Anatomy, Faculty for Biology and Preclinical Medicine, University of Regensburg, Universitätsstraße 31, 93053 Regensburg, Germany

**Keywords:** adipose-derived stem cells, mesenchymal stem cells, chorioallantoic membrane, CAM assay, wound healing, tissue regeneration, tissue engineering, scaffolds, biocompatibility

## Abstract

With a history of more than 100 years of different applications in various scientific fields, the chicken chorioallantoic membrane (CAM) assay has proven itself to be an exceptional scientific model that meets the requirements of the replacement, reduction, and refinement principle (3R principle). As one of three extraembryonic avian membranes, the CAM is responsible for fetal respiration, metabolism, and protection. The model provides a unique constellation of immunological, vascular, and extracellular properties while being affordable and reliable at the same time. It can be utilized for research purposes in cancer biology, angiogenesis, virology, and toxicology and has recently been used for biochemistry, pharmaceutical research, and stem cell biology. Stem cells and, in particular, mesenchymal stem cells derived from adipose tissue (ADSCs) are emerging subjects for novel therapeutic strategies in the fields of tissue regeneration and personalized medicine. Because of their easy accessibility, differentiation profile, immunomodulatory properties, and cytokine repertoire, ADSCs have already been established for different preclinical applications in the files mentioned above. In this review, we aim to highlight and identify some of the cross-sections for the potential utilization of the CAM model for ADSC studies with a focus on wound healing and tissue engineering, as well as oncological research, e.g., sarcomas. Hereby, the focus lies on the combination of existing evidence and experience of such intersections with a potential utilization of the CAM model for further research on ADSCs.

## 1. Introduction

An in-depth look into avian embryology may help pursue new insights into evolutionary biology and deliver practical knowledge on how to further utilize pre-existing physiological structures of a given species within a scientific framework. Starting with the fourth incubation day after a fusion of the avian allantois with the chorion, a new respiratory organ of a chick embryo is formed—the chorioallantoic membrane (CAM). Surrounding the avian embryo, the CAM as an organ plays a crucial role in its respiration and metabolism [1,2,3]. Notwithstanding, the CAM model has been actively investigated as an alternative in vivo model and first reported to be used in the studies of avian sarcomas by Rous and Murphy in 1911, given its naturally insufficient immunocompetence, high vascularization, and affordability [4,5,6,7]. The chorioallantoic membrane model can be regarded as an in vivo model that incorporates the replacement, reduction, and refinement principle (3R principle) of animal experiments [8]. Unlike other in vivo models, the CAM assay offers a reasonable compromise in terms of its utilization since it does not possess sufficient innervation up to the 17th day of ontogenesis and, thus, underlies no ethical restrictions [9]. Especially because of the above-mentioned high degree of vascularization, the CAM assay is often used in tumor research, i.e., epithelial-to-mesenchymal transition (EMT) and metastasis studies [7,10,11,12]. Further experiments with the CAM have covered a wide range of applications in virology, angiogenesis, tissue engineering, biomedical drug, and stem cell research [3,5,13,14,15,16,17,18].

In the context of stem cell research and tissue engineering, one should note the role of mesenchymal stem cells (MSCs), which are by far the most extensively studied cell type, particularly because of their angiogenic potential and overall accessibility [19]. MSCs are multipotent stromal cells that can be isolated from various tissues, including the umbilical cord, placenta, bone marrow, and adipose tissue [19,20,21,22,23]. Based on the available evidence, MSCs are also involved in the EMT within the tumor microenvironment and can be shifted into the epithelial lineage in the presence of hypoxia [24,25,26]. Hence, the CAM assay may help gain additional knowledge on the cross-talk between MSCs and cancer cells regarding angiogenesis and EMT since it represents a possible alternative to organoids and 3D cultures.

A particularly interesting subpopulation of MSC are adipose-tissue-derived stem cells (ADSCs) because of their accessibility from human lean or obese fat tissue and a versatile linage potential. Adipose tissue contains the highest ratio of MSCs per volume, as reported to date [27,28,29,30]. First identified in 2001, ADSCs have already gained clinical implementation, e.g., in lipofilling, given their potential differentiation into adipogenic, osteogenic, chondrogenic, myogenic, and endothelial lineage [20,21,22,29,31,32,33]. Further, ADSCs are also reported to influence wound healing and regeneration positively, so they may represent a potential source for tissue engineering [34,35].

In this review, we aim to investigate the utilization, potential restrictions, and future directions of the CAM assay within ADSC research. MSCs generally demonstrate an investigational profile that can be genuinely directly or indirectly assessed using the CAM model. The chorioallantoic membrane can offer a unique and affordable playground for investigating cell–cell and cell–matrix interactions, angiogenesis and vasculogenesis, immunological interactions, and tissue engineering. Finally, continuing the ongoing research on such transitional models as the CAM is important, considering the support of the 3R principle [8].

## 2. Available CAM Models and Embryology

Chicken embryo development from fertilization to egg hatching takes approximately 21 days. The first 25 h of the development of the chick were first coherently described by Eyal-Giladi and Kochav et al. and mainly include stages from cell cleavage to the formation of primitive streak [36]. Starting with the formation of the area pellucida and evolving into the demarcation of epi-, meso-, and hypoblast, Eyal-Giladi and Kochav stages XI–IV correspond to the avian developmental stages 1–2 described by Hamburger and Hamilton [36,37,38]. Besides intraembryonic circulation, development of the avian embryo also relies on the formation of extraembryonic vessels and membranes, such as the yolk sack, chorioallantois, and amnion. The chorioallantoic membrane as a new organ is formed by the fusion of the allantois with the chorion around developmental day 3.5 or stage 18 of Hamburger and Hamilton [37,39,40]. This process has been shown to involve the epithelial-to-mesenchymal transition of the chorion and allantois mesothelium [41]. Mesenchymal arterioles and venules promote the subsequent vascularization of the CAM in the ectoderm’s close vicinity, forming a new plexus from days 8 to 14 of ontogenesis. The whole process of vascularization is mainly mediated by the fibroblast growth factor 2 (FGF-2), shear stress, and vascular endothelial growth factor-A (VEGF-A) [3,42,43]. Moreover, Ribatti et al. reported distinct peaks of FGF-2 and VEGF-A levels at specific developmental stages of the avian embryo [44]. The physiological functions of the CAM, as an avian equivalent of mammal placenta, include primarily respiration and metabolism [45]. Further, increasing calcium demand for the ossification of the developing embryo is also partially covered by the ion transport from the eggshell via the CAM owing to its dense capillary network in the direct proximity of the shell [40,46]. Proteomic analyses of the CAM during its development have confirmed its functions and gained new insights into gas exchange, Ca^2+^ transportation, and defense against pathogens and luminal toxins [47]. The formation of the CAM is fully finished by day 18 of ontogenesis, showing a formed vascular plexus surrounding the avian embryo [1,40,48]. 

To date, various established protocols for the utilization of the CAM assay differ depending on the investigational field: tissue engineering, oncology, immunology, and angiogenesis. A major, fundamental difference among most of them relates to the culturing procedure: in ovo vs. ex ovo assays. In ovo assay describes an approach when experiments and manipulations with the CAM are carried out in the whole fertilized egg by the placement of a window in the egg shell, so access to the CAM can be ensured. In this way, major parts of the CAM are sealed beneath the eggshell, requiring fewer cultivating precautions and improving the avian embryo’s survival [3,39]. In ovo cultivation is considered more affordable, reproducible, and reliable [49]. On the contrary, an ex ovo assay describes a procedure of explantation of the avian embryo with adherent CAM and (parts of) the yolk sack onto a petri dish or other container prior to the time-point of the CAM’s adhesion to the eggshell [50,51]. Even though ex ovo protocols offer greater access to the CAM ensuing ancillary quantification methods, it reportedly happens at the cost of the long-term viability of the embryo [3,49]. The choice of a specific approach is mainly dependent on the experimental design. It should not be biased, considering the overall comparable cultivation requirements and availability of the CAM models derived from other species, especially Japanese quail [52]. 

## 3. Wound Healing

**Figure 1:**
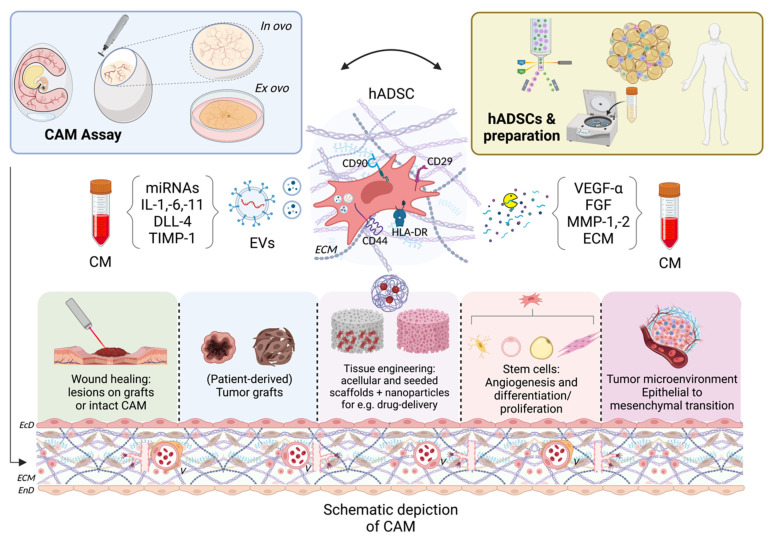
Schematic illustration of existing and potential applications of the chorioallantoic membrane (CAM) assay in ADSC research. Cells can be either isolated from human donors or obtained commercially. After a certain pre-treatment they can be grafted onto the available in ovo or ex ovo CAM models in order to investigate wound healing, tumor (anti-)angiogenesis and its microenvironment, (bio-)scaffolds, and stem cell proliferation and/or differentiation. Therefore, a human adipose-tissue-derived stem cell (hADSC) exemplarily positive for cluster of differentiation 29 (CD29), CD44, CD90, and Human Leukocyte Antigen DR isotype (HLA-DR) is depicted with its secretome, i.e., extracellular vesicles (EVs) and paracrine products, e.g., extracellular-matrix (ECM) and matrix metalloproteases (MMPs), which can be isolated and added onto the CAM as a conditioned medium (CM). The CAM structure is depicted with its outer and inner sheeting consisting of ectodermal (EcD) and endodermal epithelium (EnD), respectively. Between those two layers rich extracellular matrix (ECM) with partially spouting vessels (V) is shown. Additional abbreviations: miRNAs—micro ribonucleic acids, IL—interleukin, DLL—Delta-like protein, TIMP—tissue inhibitor of metalloproteinases, VEGF—vascular endothelial growth factor, FGF—fibroblast growth factor. Created with BioRender.com ^®^(accessed 7 February 2023).

Wound healing is a complex regenerative process modeled in vivo and in vitro for research purposes. According to some reports, a pooled general prevalence of chronic wounds of mixed etiology accounts for ~2.21 cases per 1000 population, whereas chronic ulcers of the lower extremity show ~1.51 cases per 1000 population [53]. In Germany, chronic wounds are responsible for a mean cost of EUR 9060 to EUR 9569 per patient per year, which makes it a considerable share of total health insurance expenditures [54,55]. Thus, investigating tools and mechanisms of accelerated wound healing is an important scientific topic. One of the simplest investigational models is represented by the so-called in vitro scratch assay, which has been actively used since 2007. In this model, cell–cell and cell–matrix interactions as well as cell migration can be followed to an extent that approximates the processes in vivo [56]. Alternative models include ECIS, Boyden chamber, barrier systems, and micro-fluid-based assays [56,57,58]. In addition to angiogenesis, tumor research, and tissue engineering, the CAM assay has also been described as a potential model for wound healing [5,7,59]. Moreover, the CAM assay should be treated with respect in this context primarily for the possibility of a depiction of angiogenesis and vascularization—the critical steps in any wound healing. Thus, Ribatti et al. succeeded in reproducing all critical events controlling the wound-healing process, including re-epithelialization, angiogenesis, formation of an inflammatory infiltrate, granulation tissue, and the extracellular matrix from mesenchyme [59]. In that study a minor injury was performed on the intact chorioallantoic membrane using a micro-knife and then wound healing was followed using a stereomicroscope, immunostainings, and measurements of angiogenesis kinetics with toluidine blue [59]. In the following study, the same method was used to determine the crucial role of FGF-2 for the observed effects in long-term wound healing mentioned previously—encouraging the growth of fibroblasts, macrophage infiltration, and angiogenesis [60]. In subsequent years, Zaugg et al. investigated phenotypic smooth muscle cell plasticity in a CAM-based wound model in response to thermal and chemical stimuli of the CAM by injecting intima- and media-like smooth muscle cells that had been transfected with the β -galactosidase gene and introducing them intravenously into the CAM [61]. Moreover, not only descriptive, but also functional studies of myofibroblasts, their invasion of a provisional matrix of fibrin/collagen, and the formation of granulation tissue have been described for the wound CAM assay [39,44,62,63]. Visualization of the complex inflammatory processes in other types of wounds should also be possible using CAM models. For example, Rezzola et al. succeeded in modeling diabetic retinopathy and investigating inflammation, including angiogenesis, in a CAM model exposed to the vitreous humor from pars plana vitrectomy of proliferative diabetic retinopathy samples [64]. In addition, the CAM method can be used to study xenogeneic tissues due to natural immunodeficiency: Carre et al. grafted murine fetal skin from laboratory Bagg albino mouse strain onto the CAM of 12-day-old chicken embryos and cultured them for 7 days [6,65]. Subsequently, after grafting, circular wounds were created with a rotating titanium sapphire laser and successively followed, opting for a promising model in regard to fetal wound healing [65]. From this point of view, theoretical modeling of human skin grafts that experience similar injuries can be modeled on the chorioallantoic membrane (Figure 1). According to previous reports, it is possible to xenograft human skin onto the CAM with a reasonable intake rate, reperfusion, and preservation of mammalian phenotype as far as confirmed by integrin expression [66]. 

A potential application of ADSCs to the CAM wound model would be interesting for several of reasons. Firstly, ADSCs can replace damaged cells because of their adipogenic and angiogenic potential and possible epithelial differentiation, expressing cytokeratins 5, 14, and 19 and integrins similar to keratinocytes that play a pivotal role in cutaneous wound healing [11,32,33,67,68]. Further, the facilitation of tissue regeneration is orchestrated by the ADSC-secreted extracellular matrix (ECM) proteins and ECM proteases, e.g., fibronectin, collagens I-IV, and matrix metalloproteinases (MMP-1,2) [69,70,71]. Transcriptomic analyses of ADSCs have revealed distinct expression profiles with upregulated ECM-coding collagen type XI alpha 1 chain gene, fibronectin 1 gene, and tubulointerstitial nephritis antigen-like-1 gene, as well as genes responsible for pathways involved in ECM processing and regulation—gremlin 1, myoferlin, and zinc finger, RAN-binding domain containing 1 [72,73,74]. 

However, there are presumably not only direct regenerative effects from ADSCs. Paracrine secretion and exosome analyses of ADSCs show a number of increased MMPs, as well as tissue inhibitor of matrix metalloproteinase-1 and transforming growth factor ß3 (TGF-ß3) from ADSCs, which prevent hyperplastic scar formation, regulate differentiation of fibroblasts, and thereby promote wound healing (Figure 1) [75,76]. Notably, most of the identified experiments in the context of CAM assay implementation in ADSC research provide data on the angiogenic properties of the secretome, i.e., conditioned media derived from this cell type. Notwithstanding the influence on the ECM, exosomes from ADSCs were found to inhibit apoptosis via the wingless-related integration site/β -catenin (*Wnt/β-catenin*) pathway as measured with Western blotting [77]. In such a way, authors have assumed an enhancement of cell proliferation, migration, and inhibition of apoptosis in the described in vitro wound model [77]. This pathway has been previously reported to contribute to wound healing by impairing apoptosis in Cre mice with a conditionally inactivated β-catenin pathway (*Catnb^tm2Kem^* knock-out mice) [78]. The increase in angiogenesis mediates further effects on wound healing by ADSCs. Human adipose-tissue-derived stem cells were shown to promote angiogenesis through proteolytic collagen remodeling of MMPs and a close interplay with sessile endothelial cells in vitro [79]. The angiogenic potential of ADSCs was first described by Rehman et al. by the secretion of VEGF, granulocyte macrophage colony-stimulating factor, hepatocyte growth factor, and TGF-α [80]. It has been subsequently confirmed by other authors based on enzyme-linked immunosorbent assays and real-time polymerase chain reaction (RT-PCR) [70,81,82]. A number of micro-ribonucleic acids (microRNA-125a, microRNA-31) from exosomes of ADSCs may also potentiate angiogenesis by inhibiting the expression of angiogenesis inhibitor delta-like protein 4, and, hence, being transferred to endothelial cells promoting angiogenesis [75,83,84]. A potential down-regulator of angiogenesis in this context is microRNA-195. Particularly for the CAM assay, it has been shown that human bone-marrow-derived MSCs electroporated with microRNA-195 show a 28% decrease (*p <* 0.01) in endothelial vessel growth [85]. On the contrary, according to some reports, the angiogenic capacity of ADSCs on the CAM model can be increased by, e.g., cryo-temperature pretreatment, electrostimulation, and specific oxygenation conditions. Zhu et al. report human ADSC spheroids after exposure to hypothermic stress to promote angiogenesis in ovo and to activate the phosphatidylinositol 3-kinase/Akt pathway with upregulation of expression of *FGFs*, hepatocyte growth factors, and other angiogenesis-related factors [86]. Similarly, autologous frozen fat grafts enriched with human ADSCs lead to greater angiogenesis, VEGF-expression, and cell survival on the chorioallantoic membrane in ovo (Table 1) [87]. A conditioned medium of electro-stimulated ADSCs led to an increased vessel density and total vessel network on the CAM with higher VEGF-A and monocyte chemoattractant protein 1 expression levels, yet it caused a decrease of the anti-angiogenic protein Serpin E1/plasminogen activator inhibitor-1 [88]. A reduction in oxidative stress appears to positively influence angiogenic capacities of ADSC-seeded scaffolds in ovo (Table 1) [89]. Lastly, hypoxic conditions are believed to increase hypoxia-inducible factor 1 alpha (HIF-1α) levels and VEGF secretion, hence, leading to increased angiogenesis [90,91]. Expression of HIF-1α by the ADSCs from human lipoaspirates was previously reported to, e.g., increase vascularization, capillary density, and survival of skin flaps in diabetic mice [92]. Increased vascularization can be achieved not only via pre-conditioned media or cell suspensions but also for the whole ADSC-based cell sheets as allo- or xenogeneic grafts, as has been shown for diabetic and for thermic wound models in vivo (Table 1) [93,94,95,96]. 

Addressing an application of the CAM assay with MSC/ADSCs, it is noteworthy to mention the potential of human MSCs to change into a non-proliferative phenotype when transplanted onto the CAM. Thus, avian MSCs gain a vasculogenic and pericyte-like fate, which implies its direct proangiogenic arrangement in response to the grafted human MSCs. However, this has been shown in a tumor CAM model only [114,115]. Further, the possibility of establishing a xenogeneic capillary network has been demonstrated for human MSCs transplanted onto the CAM. In the study of Cosma et al., human mesenchymal stem cells switched to a CD44-negative, endothelial, non-proliferative phenotype, whereas avian MSCs organized themselves into vasculogenic, capillary-like structures obtaining avian CD34- and smooth-muscle-antigen-positivity [115]. Similarly, Strassburg et al. reported human ADSCs in a co-culture with human endothelial cells to enhance a formation of human CD31-positive capillary-like structures on the CAM model (Table 1) [101]. Conditioned media from co-cultures of growth-arrested ADSCs with endothelial cells can improve cell proliferation, migration, and angiogenesis in vitro and in ovo (Table 1) [112]. Additionally to the previously described contributions, ADSCs release a number of cytokines, e.g., interleukins 6, 8, and 11 (IL-6, IL-8, IL-11), and tumor necrosis factor-α (TNF-α) [81,116,117]. This in turn creates an immunomodulatory milieu in the close vicinity of ADSCs that inhibits the immunologic response and might have a beneficial influence on the wound-healing process [118]. Interestingly, these levels of, e.g., IL-6 produced by ADSCs also seem to be dependent on the oxygenation within the tissue, so hypoxic conditions with an increased level of HIF-1α exposure might foster the proliferation and differentiation of fibroblasts [119,120,121]. Even though avian interleukins seem to be detected at different developmental stages, e.g., IL-6 could be identified at later stages after day 18, some studies have shown an enhanced angiogenic response towards some of the externally added interleukins on the CAM [122,123]. Thus, administration of recombinant IL-6 or human-monocyte-derived IL-1β results in an angiogenic response on the CAM and in endothelial cell proliferation of human umbilical vein endothelial cells (HUVECs) on Matrigel [124,125]. However, the vasculogenic effects of the ADSCs seem to be a prerogative of innate stem cells. The secretome of senescent human ADSCs was reported to inhibit angiogenesis in the CAM model, potentially due to the impaired paracrine cell communication and downregulation of a number of genes involved in vasculogenesis (Table 1) [109]. 

The chorioallantoic membrane assay and ADSCs share a mutual investigational profile in wound healing and tissue regeneration that should be further explored in future studies. Since 2001, the Food and Drug Administration of the United States has approved the CAM assay for preclinical evaluation of drugs to be approved for the treatment of burn wounds and chronic skin ulcers [44,126]. In that regard, ADSCs as injections or scaffold-based constructs have also been reported to promote wound healing and regeneration after thermic injuries [127,128,129,130]. Clinically, there are many ongoing studies evaluating the role of ADSCs in the facilitation of regeneration. For example, JOINSTEM, a phase-III study (NCT04427930), investigates a possible application of autologous ADSCs in patients with knee osteoarthritis. Other clinical applications cover a wide range of options from knee or hip osteoarthritis (NCT03467919, NCT03608579), spinal cord injuries (NCT02917291), corneal dystrophies (NCT05279157), partial-thickness rotator cuff tears (NCT03752827), up to chronic concussive syndrome (NCT04744051) and subcutaneous fat grafting (NCT05079243). In this way, the CAM assay may help explore new horizons of therapeutic ADSC utilization as an alternative in vivo model for specific hypotheses.

## 4. Primary Cell Tissues, Cultures, and Sarcoma Research

One of the reasons for the utilization of the CAM model lies in its comparability with xenograft tissues and cells, notably also with primary cell cultures. Primary cell culture is a way of culturing freshly acquired cells without previous immortalization in vitro. Regardless of their higher maintenance costs based on their slower growth, special culture conditions, finite lifespan, and lower senescence, primary cell cultures represent higher biological relevance because of their high similarity to the tissue of origin. Thus, experiments with primary cells and tissues are considered more valid and representative [131]. This way, the CAM assay could represent a robust in vivo xenograft culture for patient tissues within a personalized therapy approach, tissue engineering, and drug testing. 

As mentioned, the first experiments studying the growth of primary xenografts (sarcoma tumors) on the CAM were performed by Rous and Murphy in 1911 [4]. In the following decades, Hurst et al. demonstrated neoplastic and normal human and rabbit tissue survivorship on the CAM model [132]. Karnofsky et al. and Dagg et al. have performed inoculation experiments with chicken sarcomas and human tumors on the CAM, where they described the histomorphological characteristics of the growth and metastatic potency of transplanted primary tissues [133,134,135]. In 1991, Shoin et al. grafted human tumor specimens from 57 resected tumors onto CAM and reported all engrafted tissues to adhere and grow on the CAM after seven days of incubation [136]. Another study on normal fat tissue vs. benign musculoskeletal tumors on the CAM was performed in 1999 by Lucarelli et al., who showed a comparable rate of increased angiogenesis in lipoma tissues from nine consecutive patients to those of the CAMs treated with FGF-2. In contrast, normal fat tissue did not provoke any additional angiogenic response [137]. 

Specifically, sarcomas are known for their problematic cultivation in vitro [138]. However, the CAM assay appears to be a robust alternative for studying the morphology and invasiveness of human sarcomas (Figure 1). Sys et al. have xenografted samples from 28 consecutive patients with musculoskeletal tumors, including 17 sarcomas, onto CAM and reported a viability rate of 42.7%, with no significant difference between benign and malignant tumors [139]. Yet, in that study, tissues varied in their viability/necrotic portion prior to engraftment onto the chorioallantoic membrane because of the explantation site (primary tumor vs. metastasis) and previous treatment history (neoadjuvant chemotherapy vs. primary resection). Therefore, viable tumors showed characteristics of their primary site based on morphology and immunohistology [139]. Further, engrafted tumors similarly differed in the vascularization and chick fibroblast invasion rate depending on their primary entity [139]. The following year in 2013, Sys et al. published their xenograft protocol for resected fresh sarcoma-derived specimens and sarcoma cell lines—a valuable tool for further research on musculoskeletal-derived tumors in the unique tumor microenvironment of the CAM [140]. This protocol was used and adapted by Guder et al. in their study of 26 patients with musculoskeletal tumors, which, after resection, were freshly grafted onto CAM as primary tissue or cell culture [141]. Subsequently, after six days of incubation, tissue and cell cultures were additionally incubated with 5-aminolevulinic acid and exposed to blue or red light to measure the tumor fluorescence or therapeutic effect of photodynamic therapy [141]. Even though the viability rate of the inoculated tissue remained comparably low at approx. 17.7%, primary cell culture samples were all viable at a 100% rate [141]. Feder et al. grafted various parts of primary osteosarcoma tissue onto CAM. Primary osteosarcoma tissue grew on several different CAM models for an extended period, and neovascularization of serial transplanted tumor parts was observed, improving the versatility of the 3D in vivo tumor model [142]. In this way, we hypothesize that further experiments with primary tissues on the CAM assay should occur, as it is a promising in vivo model for sarcoma research [17]. 

Adipose-tissue-derived stem cells have also taken their rightful place in oncological research. Alongside a potential role in the pathogenesis of breast, ovarian, and prostate cancers, ADSCs have been found to be involved in the pathogenesis of sarcomas, especially osteosarcomas. On the one hand, there is yet weak evidence that ADSCs and their close relatives MSCs can directly (de-)differentiate into sarcoma cells (specifically, leiomyosarcoma) through loss-of-function mutations such as p53 deficiency [143,144,145]. Moreover, other authors hypothesize karyotypic changes in MSCs at varying differentiation stages (aneuplodization, genomic losses) as a direct mechanism of sarcoma formation [146]. Conversely, MSCs can switch into a reactive phenotype in the close vicinity of osteosarcomas and are therefore referred to as cancer-associated fibroblasts [147,148]. One of the possible interactive mechanisms in the tumor cross-talk with MSCs and ADSCs is a so-called reverse Warburg effect—a “reversed” hallmark of cancer [149,150,151,152]. Bonucelli et al. were the first to investigate the inflection of MSC derived from adipose tissue in osteosarcoma metabolic reprogramming [152]. In that study, ADSCs were shown to undergo oxidative stress due to the tumor’s production of reactive oxygen species and, hence, shift towards aerobic glycolysis with an increased lactate production accelerating tumor cell migration [152]. The signal transducers and activators of the transcription-3/Interleukin-6 (STAT3/IL-6) pathway was hypothesized by Bonucelli et al. to be responsible for these findings. They referred to a previous study in which STAT3 was inhibited in osteosarcoma cell lines in MSC-preconditioned medium by short interfering RNA or AG490 (Janus kinase/STAT inhibitor), resulting in a decrease in cell invasion, proliferation, and migration rates [152,153]. Interestingly, a later study identified ADSCs to interact with osteosarcoma cells via the STAT3 pathway. This pathway, which physiologically mediates the effects of growth factors and interleukins, could also be responsible for a metabolic shift in cells within the tumor microenvironment [154,155]. On the other hand, even when exposed to chemotherapeutical agents, ADSCs seem to maintain their capacity to promote tumor invasion and pro-metastatic effects on the CAM model, as has been shown by Plava et al. for breast cancer [156]. 

As mentioned in the chapter about wound healing, paracrine cell communication is one of the critical characteristics of ADSCs’ contribution towards the regenerative process. From this point of view, exosomes from osteosarcoma may also be responsible for the engagement of hostile MSCs/ADSCs in their promotion of angiogenesis, metastasis, and cell proliferation [157,158]. However, there are direct and indirect effects of MSCs and tumor cells owing to cell–cell communication within the tumor microenvironment, such as epigenetic reprogramming. Hence, Mannerström et al. demonstrated that human ADSCs exposed to osteosarcoma-isolated exosomes inherited epigenetic alterations with global long interspersed element hypomethylation-1 [159]. In that way, ADSCs treated with sarcoma-derived exosomes demonstrated tumor-like perturbations with higher expression rates of genes critical for ECM remodeling, angiogenesis, and cell invasion (*MMP1*, *VEGF-A*, and intercellular adhesion molecule-1, respectively) [159]. Thus, the tumor “prepared” a hostile milieu, i.e., microenvironment, for its further spreading and growth. A vice versa response transmitted by exosomes from MSCs towards the tumor was reported for bone-marrow-derived MSCs that contributed to tumor progression. A study of Zhu et al. demonstrated that *VEGF* and C-X-C motif chemokine receptor 4 expression in human gastric carcinoma cells are increased by MSC exosomes via mitogen-activated kinases [113]. Similarly, ADSC-derived exosomes can contribute to the invasiveness, spreading, and proliferation of osteosarcoma cells via upregulation of the collagen beta(1-O)galactosyltransferase 2, a gene responsible for the enzymatic glycosylation of collagen in the endoplasmic reticulum [160,161]. 

On the other hand, ADSCs seem to play a role in tumor formation and spread. Some reports postulate the therapeutic effects of using ADSCs in sarcoma research (Figure 1). These effects and results from the studies are still conflicting. It seems there is an interconnection between the cell cycle stage of the tumor and a possible impact from injected ADSCs, also depending on the application form—intratumor injection vs. s.c. injection [162,163]. Further, Lee et al. demonstrated different responses towards ADSC injection depending on its concentration: when ADSCs were mixed with osteosarcoma cells in low proportions of 5–15%, they showed a modifiable inhibitory impact on cancer progression, but larger concentrations (25%) might encourage tumor development [162,164]. Additionally, MSC-derived exosomes have already found their implementation as drug-delivery vehicles, anti-cancer therapeutics, immunomodulators, and facilitators of regeneration [162,165,166].

In conclusion, the final role of MSCs, especially ADSCs, in the oncogenesis or cancer progression is yet to be determined. The chorioallantoic membrane assay offers a unique tool for refining available primary tumor and stem cell culture protocols, where different snapshots of the cross-talk between tumor and, e.g., ADSCs can be followed directly or indirectly. Considering that only insufficient data on the utilization of extracellular vesicles explicitly derived from ADSCs in the pathogenesis and (targeted) therapy of sarcomas exist, it is essential to continue the ongoing research.

## 5. Tissue Engineering

The chorioallantoic membrane as a highly vascularized respiratory and metabolic organ has various applications in tissue engineering research, reaching from the analyses of angiogenesis and neovascularization of biocompatible materials to regeneration and differentiation of allo-/xenografted tissues. Tissue engineering itself comprises the replacement or repair of damaged tissues with the use of artificial or semi-artificial substitutes. The most popular approach so far has been scaffold-based tissue engineering, i.e., delivery of (a-)cellular matrices to tissues in order to facilitate their regeneration. For this purpose, scaffolds have to provide a few main characteristics that make them desirable for research and pre-clinical establishment: low immunogenicity, biocompatibility, close resemblance of physiological structures and mechanical properties, e.g., ECM, and low toxicity. However, these requirements are expanded by the functional spectrum of the cell/tissue type of interest. Therefore, a “perfectly” engineered skin scaffold would ideally represent the functionality of all three skin layers with the epidermis, cutis, subcutaneous fat and ability to vascularize; a scaffold for peripheral neuroregeneration would promote axonal growth and would be susceptible to electrical stimulation; a bone scaffold would be able to mineralize and demineralize, i.e., bone-remodeling, etc. An approach for the combined functional repertoire of scaffolds is the so-called multifunctional bio-scaffolds, which can be decellularized and reassemble ECM properties with additional functions [167,168]. One of the most essential and challenging milestones in tissue engineering is a recreation of vasculature within the scaffold or organoid [169,170]. In the case of acellular scaffolds, de novo vascularization occurs as an answer to growth factors or other functional molecules present on the transplanted ECM structure [171]. The benefits of such a solution include low immunogenicity due to the reduction in antigen burden within the scaffold, biocompatibility, and architectural pre-requisite formation for cell migration and differentiation. Such scaffolds can be either seeded with cells or specific bioactive molecules in order to promote a desired process or solely transplanted onto the damaged tissue without previous seeding. 

One of the first study groups to describe the reaction of the CAM towards transplanted biomaterials was D’Arcy and Howard et al. in 1967, who placed a sterilized filter paper on top of the CAM, looking for an inflammatory response [172]. After years of research, Zwadlo-Klarwasser et al. employed the CAM assay to systematically examine the reactivity of materials used or intended for use as short- or long-term implants based on these preliminary findings [173]. It has been shown that the chemical composition and architecture of biomaterials influence the angiogenic activity and inflammatory response of the CAM: reduced angiogenesis in materials with smooth surfaces (e.g., polyurethane, polyvinyl chloride) and vice versa (collagen-based materials); lower anti-angiogenesis in materials with negatively charged particles (plasticizer diethylhexyl-phthalate or triethylhexyl-trimellitate) than in positively charged ones; symmetrical structure resulting in a lower inflammatory response [173]. This led to a hypothesis that the material properties prior to transplantation can induce a predictable impact on the inflammation and vascularization of the grafted construct. For example, a more extensive material porosity can provide significantly higher vascularization and cell invasion rates than less-porous materials [174,175]. Similar findings were made for ADSCs seeded onto different materials (Table 1) [102]. Thus, exposure of ADSCs to hydrogels of low stiffness (0.15 kPa) may have led to an altered redox metabolism with implications for its secretome since its conditioned medium was reported to increase the angiogenesis and proliferation of HUVECs ex ovo (Table 1) [108]. Oates et al. compared scaffolds with different pore sizes in terms of inflammatory response and angiogenic capacity of the chorioallantoic membrane, further utilizing PCR with primers for TNF-a in order to quantify the inflammatory response [176]. Materials with greater sizes of pores were demonstrated to have a weaker inflammatory reaction in terms of TNF-a secretion by the hostile CAM and a slightly increased angiogenesis in response to scaffolds with a 90% porosity [176]. In line with these findings, Samourides et al. found that polyglycerol sebacate urethane (PGSU) scaffolds with larger porosity and pore size distribution (PGSU-5%) induce a substantial fraction of collagen formation and prompt angiogenesis in addition to deep tissue ingrowth [177]. Using multilayered agent-based model simulation, Artel et al. demonstrated that higher pore diameters of around 160 to 270 µm promoted angiogenesis across the scaffold [178]. Scaffold durability similarly provides clues about the in vivo tissue response. Especially, tissue spread based on cell migration and invasion seems to be more efficient in tissues with a higher cross-linking rate [63]. Even though an induced angiogenesis rate appears to occur at the cost of lower tissue contraction rate, some authors have reported increased cross-linking of scaffolds to increase vascularization and cell proliferation rate within the scaffold or in close vicinity to the scaffold [63,179,180,181,182]. It has been further reported that transglutaminase-treated scaffolds may also have a positive effect on angiogenesis, even in materials conventionally believed to demonstrate anti-angiogenic properties, e.g., the amniotic membrane [183]. However, the extent of cross-linking may be limited due to a potential formation of cytotoxic byproducts such as degradation products and unreactive monomers, at least in synthetic materials [184]. 

When addressing angiogenesis and immunomodulation, adipose-tissue-derived stem cells cannot be ignored, particularly not in the tissue engineering context. Because of their high proliferative potential and the expression of genes crucial for angiogenesis and paracrine cell communication (e.g., *VEGF*, *BFGF*, or leptin), ADSCs are an important tool for scaffold-based tissue engineering. Borges et al. were some of the first investigators who demonstrated a solid angiogenic potential of ADSCs in combination with fibrin matrices on the CAM, presumably owing to the secretion of VEGF and basic FGF (Table 1) [97]. Whether or not ADSCs can differentiate into endothelial cells on the CAM model remains questionable. However, co-culture experiments appear to confirm a de novo formation of capillary-like structures on the CAM in combination with endothelial or human-umbilical-cord derived stem cells (Table 1) [101]. Hereby, adipose-tissue-derived stem cells in co-culture with endothelial cells seem to cause more efficient angiogenesis both in vitro and in vivo compared to HUVECs or other MSCs [185]. Notably, it has yet to be tested what differentiation potential primary human ADSCs can have in ovo and whether the delivery of scaffolds can facilitate this process onto the CAM. A significant finding and, thereby, a challenge is to determine how different cell types react to materials of varying architecture and composition. For example, with the aid of the 3D polylactic acid scaffolds, ADSC culture was given a useful environment that promoted cell cycle progression while also enabling the preservation of their undifferentiated form [186]. On the other hand, endothelial progenitors under the same conditions showed reduced proliferation and an altered immunophenotype [186]. Adipose-tissue-derived stem cells from human lipoaspirates cultured with FGF and VEGF showed a higher endotheliogenesis and proliferation rate when seeded onto scaffolds of small intestinal mucosa [187]. A hypothesized mechanism behind this is an activation of the Src pathway in response to FGF-2, a tyrosine kinase crucial for controlling how eukaryotic cells develop and differentiate [188,189]. Sequencing studies of CD34+ ADSCs undergoing endotheliogenesis have identified a number of enriched microRNAs, such as microRNA-181a, which has been reported to induce angiogenesis via the Src pathway in colorectal cancer [190,191]. Yet, there are also other effects, e.g., physical effects that can promote angiogenesis. Recent studies have reported an increased vascularization rate of ADSCs (in co-culture with HUVECs on the CAM) in response to irradiation with red light (photo-bio-modulation), possibly due to increased mitochondrial activity, nitrogen-oxide-based enhancement of tissue perfusion, and inhibition of inflammatory response as a result of biological leukotriene B4 inactivation [192,193,194]. 

Practically, the CAM assay is widely used to test the angiogenic properties of biomaterials (Figure 1, Table 1). When evaluating angiogenesis, decellularized scaffolds appear to be a proper way to investigate de novo vascularization on the CAM. Ribatti and colleagues have studied a wide range of acellular scaffolds obtained from different organs, demonstrating a response equivalent to FGF-2-induced angiogenesis [169,195,196,197,198,199]. However, efficient tissue-specific engineering often relies on the additional seeding of these matrices with a desired cell type, mostly with stem cells of different potency or their bioactive molecules. From this point of view, ADSCs can be used to populate initially acellular scaffolds for regenerative purposes. It can, in turn, reduce problematic steps in the process of scaffold intake owing to cell-specific properties. For example, Perea-Gil et al. used decellularized human peri- and myocardial scaffolds seeded with porcine ADSCs in the in vivo model of myocardial infarction (MI) and reported more diminutive MI size as well as better left ventricular ejection fractions and end-systolic volumes for re-cellularized scaffolds compared to acellular ones [167,200]. In such a setting, human ADSCs can also function as drug-delivery systems (e.g., statin-loaded nanoparticles), showing similar positive effects on the infarcted myocardium and further promoting angiogenesis while reducing inflammation [167,201]. There can be a fairly broad spectrum of applications when addressing scaffolds in the cross-section of CAM assays and ADSCs (Figure 1). Watchararot et al. showed biocompatibility of silk fibroin scaffolds on the CAM and more rapid angiogenesis of these scaffolds when seeded with ADSCs [111]. Silk fibers isolated from *Bombyx mori* silkworm demonstrate appealing future uses as biomechanical materials due to their distinctive mechanical and biological characteristics [202]. Silk-based scaffolds induce a greater angiogenic response than monofilament, polyethylene terephthalate scaffolds in ovo and promote osteogenic differentiation of human ADSCs in vitro [203]. Other porous scaffolds are represented by chitosan, poly-lactic-to-glycolic (PLGA), hyaluronic acid-based scaffolds and polycaprolactone (PCL) nanofibers, etc. Cheng et al. reported a dramatically accelerated capillary development in the CAM experiment and endothelial cell tube formation in the mouse wound model in vivo for ADSC-seeded chitosan hydrogel [104]. Further, it has been shown that those scaffolds had greater porosity complemented by a greater release of cells and VEGF when ADSCs were enclosed (Table 1) [103]. Buschmann et al. found that ADSC-seeded electrospun PLGA/amorphous calcium phosphate nano-scaffolds induced sustained cell proliferation with a phenotype switch towards osteogenesis, graft invasion, and avian angiogenesis on the CAM model (Table 1) [98]. Interestingly, other authors reported a higher mineralization and cell invasion rate of cell-free, ADSC-derived 3D microtissue secretome-seeded collagen scaffolds on the CAM (Table 1) [110]. Handel et al. showed a significant induction of vascularization for the 45S5-Bioglass^®^-based 3D-scaffolds seeded with human ADSCs in the CAM, presumably due to VEGF secretion (Table 1) [100]. Additionally, seeding of 17-β-estradiol-releasing polyurethane scaffolds with human ADSCs showed higher ECM production, enhanced angiogenic potential, and good tissue integration on the CAM (Table 1) [105]. Lastly, ADSCs are reported to form biomaterial-free structures—“a living scaffold” consisting of fiberoids—to integrate within the tissue on the CAM and to promote a significant angiogenic reaction in combination with HUVECs (Table 1) [107]. Interestingly, another type of ADSC-derived cells, beige cells, which are a rather underestimated cell type in tissue engineering, can be further utilized as angiogenetic facilitators in ovo, as has been reported for histone-based bio-scaffolds by Di Somma et al. (Table 1) [106].

The fusion of different scaffold types may deliberately enhance the desired functions of the material. Thereby, human pediatric ADSCs seeded onto a biodegradable nanocomposite polymer, polyhedral oligomeric silsesquioxane poly(ε-caprolactone-urea) urethane (POSS-PCL), were shown to differentiate and proliferate into specific mesenchymal lineages in vitro and to be biocompatible on the CAM [103]. Therefore, ADSCs’ epithelial (CK18 and zona occludens antigen-1 positive), chondrogenic (collagen-II), osteogenic (mineralization), and adipogenic (oil-droplets) differentiation was reported in vitro for POSS-PCL scaffolds (Table 1) [103]. In general, higher mineralization and, hence, higher activity of alkaline phosphatase (ALP) was reported to induce osteogenic differentiation of ADSCs in response to polypyrrole-coated polylactide scaffolds or bioactive silicate nanoplatelets [204,205,206]. Osteogenic or chondrogenic differentiation of MSCs, particularly of ADSCs, is an essential topic in bone regeneration research (Table 1) [99]. In this context, a distinct subset of genes and transcription factors has been identified that are associated with such a fate switch. One of them—core-binding alpha factor-1 (*CBFA1*/*RUNX2*)—is a prerequisite for the downstream activation of *Wnt* and bone morphogenic protein (BMP) signaling pathways within the osteoblast commitment [207,208]. For example, Zuk et al. and Liu et al. have measured elevated expression levels of *CBFA1* after exposure of human ADSCs to an osteogenic medium [22,209]. The above-mentioned synthetic polymers (e.g., PLGA, PCL, poly-L-lactic acid scaffolds) have also been shown to induce osteogenic differentiation of human ADSCs [208]. Notably, the differentiation of ADSCs was mainly studied in vitro in those studies. It is hard to sufficiently investigate the stem cell differentiation process within the chorioallantoic membrane model given a limited experimental time window of only a few days. Notwithstanding the CAM model, xenogeneic experiments with avian embryos and human ADSCs can be carried out by engraftment of these cells directly into the avian embryo. For example, it was reported that human ADSC spheroids grafted into the presumptive presomitic mesoderm of chicken embryos adopt a perineural niche in vivo, and a minority of them obtain fates typical of neural-crest derivatives [210].

On the other hand, sessile avian MSCs were shown to obtain Cbfa-1+, BMP-4+, and osteonectin-positive osteoprogenitor and osteoblastic phenotypes in response to hyaluronic acid/bone substitute complex implanted onto CAM [211]. A study design has yet to be changed to assess the proliferation or differentiation of stem cells on CAM. Potentially, experiment expansion in terms of a transfer of pre-formed or pre-differentiated tissue and cell masses onto CAM from in vitro is still possible, as has been shown for, e.g., placenta-derived MSCs and Wharton’s jelly-derived MSCs [212,213]. Further, with the advances in quantification techniques, e.g., single-cell RNA sequencing, it is theoretically possible to detect early transcriptomic changes of (xeno-)grafted stem cells on the CAM, which may predict a fate change of ADSCs towards one of the directions of the trilineage differentiation. 

In conclusion, the CAM assay is preferably used to study the angiogenic properties of natural and synthetic scaffolds that can also be seeded with human ADSCs to enhance regeneration of the tissue of interest. Nevertheless, there is potential for further adaptations and modifications of pre-existing CAM protocols that can allow a closer examination of snapshot-like, specific stages of stem cell proliferation, differentiation, or invasion of the grafted tissue in the regenerative context. We speculate that the CAM assay can provide a bio-reactive “chamber” for the further investigation of human ADSC-seeded scaffolds, especially with the assistance of novel quantification techniques and read-outs.

## 6. Conclusions

The benefits of the CAM model as a 3D in vivo model include relative immunodeficiency as well as the potential investigation of xenogeneic cells or tissues, primary cell cultures, and scaffold testing (3D cultures and patient-derived xenografts), which make it one of the most desired models for (anti-)angiogenesis studies. This makes the model especially interesting for the aforementioned potential applications. Further, the CAM assay appears to be reliable, cost-effective, and easy to use [49]. The CAM enables the assessment of a multitude of variables and includes most of the known in vitro and some of the in vivo techniques, as well as artificial-intelligence-based quantifications, MRI, CT, and even PET scans [3,46,192,214,215,216]. In general, the utilization of the CAM model complies with the 3R principles despite the fact that there are different regulations concerning the time-point of experiment withdrawal depending on the specific country [8]. Moreover, the choice of a specific point in time (during the development of the CAM) for an experimental intervention depends on the hypothesis tested. It can differ according to the application field, which may present an obstacle to overcome. Furthermore, a relevant level of similarity has been reported for the avian and human genomes, but the differences could play a crucial role in processes that remain unclear or not yet explored, especially at the cellular and molecular levels [217,218]. This implies that any results generated with the CAM assay that include the usage of human biomaterials (e.g., cells and tissues) have to be treated with caution. The CAM assay still remains a primarily pre-clinical model, and the possibilities for translational validations between different species are limited. Moreover, the previously mentioned short incubation period could also become a limitation in the experimental setting regarding the differentiation potential of stem cells or genomic implications of drugs or bioactive molecules. In summary, the CAM model should be considered as a transitional model for the experimental steps between available in vitro and in vivo models and not as a substitute for other in vivo models.

## Figures and Tables

**Table 1:** Overview of studies utilizing CAM assay in the context of adipose stem cell research.

Author and Year	CAM Assay	ADSC Origin	Context of Utilization	Main Findings	Ref.
Borges et al. (2006)	In ovo	Human subcutaneous tissue derived from surgery	Vascularization and angiogenic effects of ADSCs in fibrin matrix	Significantly increased angiogenesis in the intervention group	[97]
Buschmann et al. (2012)	In ovo	Lipoaspirated, pretreated human cells	Angiogenic potential of ADSC-seeded PLGA/a-CaP electrospun scaffolds	Homogeneous vessel distribution within the tubes	[98]
Guasti et al. (2013)	In ovo	Lipoaspirated, pretreated paediatric human cells	Vascular response to human ADSCs-seeded POSS-PCU scaffolds and cell survival	Successful vascularization and presence of ADSCs within the scaffold	[99]
Handel et al. (2013)	In ovo	Lipoaspirated, pretreated human cells	Angiogenic effects of ADSC-seeded 45S5-Bioglass-Based 3D scaffolds	Significantly increased angiogenesis in the intervention group compared to human-fibroblast-seeded scaffolds	[100]
Strassburg et al. (2013)	In ovo	Human subcutaneous tissue derived from surgery	Angiogenic effects of ADSCs in co-culture with endothelial cell and HUVEC spheroids in fibrin matrix	Significantly increased angiogenesis in the intervention group with HUVECs	[101]
Wahl et al. (2015)	Ex ovo	Lipoaspirated, pretreated human cells	Angiogenic effects of CM from ADSC-seeded chitosan, fibrin, bovine collagen, and decellularized porcine dermis scaffolds	Significantly increased angiogenesis for CM from seeded COL/GAG matrices	[102]
New et al. (2016)	Both	Lipoaspirated, pretreated paediatric human cells	Angiogenesis and compatibility of ADSC-seeded nanoscaffold composites	Proof-of-concept for the intervention group in terms of in vivo biocompatibility, angiogenesis, and vascularization	[103]
Cheng et al. (2017)	In ovo	Human subcutaneous tissue derived from abdominoplasty	Angiogenic effects of ADSC-blended collagen/chitosan hydrogels	Significantly increased angiogenesis in the intervention group	[104]
Shafaat et al. (2017)	Ex ovo	Human subcutaneous-fat-tissue-derived	Angiogenic effects of ADSC-seeded estradiol-releasing PU scaffolds	Significantly increased angiogenesis in the intervention group	[105]
				Increased density of ECM in the intervention group	
Beugels et al. (2019)	In ovo	Lipoaspirated, pretreated, Single-donor human cells	Angiogenic effects of ADSC- derived secretome post-electrostimulation	Significantly increased angiogenesis in the intervention group	[88]
Di Somma et al. (2019)	In ovo	Lipoaspirated, pretreated human cells	Angiogenic effects of ADSC-derived beige cells	Significantly increased angiogenesis in the intervention group	[106]
Sousa et al. (2019)	In ovo	Human ADSCs (ATCC)	Angiogenic effects of ADSC- derived cell-fibers	Significantly increased angiogenesis in the intervention group with HUVECs	[107]
Teo et al. (2019)	In ovo	Lipoaspirated, pretreated human cells	Angiogenic effects of ADSCs equipped with antioxidizing particles exposed to H_2_O_2_	Significantly increased angiogenesis for cells tethered with particles loading EGCG and MnO_2_ nanocatalysts	[89]
Yang et al. (2019)	Ex ovo	*hTERT* immortalized ADMSCs	Angiogenic effect of CM from ADSCs exposed to low-stiffness hydrogel	Significantly increased EC proliferation, migration, and angiogenesis in the intervention group	[108]
Ratushnyy et al. (2020)	In ovo	Non-senescent and senescent (long-term cultivated), lipoaspirated human cells	Comparison of angiogenic effects of CM of both groups	Significantly decreased angiogenesis in the senescent group	[109]
Otto et al. (2021)	In ovo	Lipoaspirated, pretreated human cells	Angiogenic effects of combinations of sc-ADSCs, 3D-MT ADSCs, and its secretome in a collagen scaffold	Significantly increased angiogenesis for sc-ADSCs	[110]
				Significantly increased COL formation for sc-ADSCs	
				Significantly increased mineralization for 3D-MT ADSC secretome	
Watchararot et al. (2021)	Ex ovo	Lipoaspirated, pretreated human cells	Angiogenic effects of ADSC-seeded vs. acellular SF scaffolds	Significantly increased angiogenesis in the intervention group at day E11	[111]
Ezdakova et al. (2022)	In ovo	*hTERT* immortalized cells (*ASC52telo*)	Angiogenic effect of CM from ADSCs and ECs co-culture	Significantly increased angiogenesis in the intervention group	[112]
Lin et al. (2022)	In ovo	Lipoaspirated, cryopreserved human cells	Angiogenic effects of cryopreservation	Significantly increased angiogenesis in the intervention group	[87]
Yu et al. (2022)	In ovo	Human subcutaneous-fat-tissue-derived	Angiogenic effects of ADSC-spheroid-integrated cell sheets	Significantly increased angiogenesis in the intervention group	[96]
Zhu et al. (2022)	In ovo	Lipoaspirated, pretreated human cells	Angiogenic effects of hypothermic pre-treatment	Significantly increased angiogenesis in the intervention group	[113]

Abbreviations: ADSC—adipose-tissue-derived stem cell; sc—single-cell; MT—microtissue; hTERT—human telomerase reverse transcriptase; EC—endothelial cell; COL—collagen; GAG—glycosaminoglycane; HUVEC—human umbilical vein endothelial cell; PLGA/a-CaP—poly-lactic-co-glycolic acid and amorphous calcium phosphate nanoparticles; POSS-PCU-polyhedral oligomeric silsesquioxane-poly(carbonate-urea)urethane, CM—conditioned medium; EGCG—epigallocatechin gallate; MnO_2_—manganese oxide; H_2_O_2_—hydrogen peroxide; E11—eleventh day of development.

## Data Availability

Not applicable.
